# A digital adaptation of the WHO’s Self-Help Plus psychological intervention to alleviate stress among community health workers: a mixed-methods evaluation of the SAMBHAV program in rural India

**DOI:** 10.1093/heapol/czaf075

**Published:** 2025-10-16

**Authors:** Ritu Shrivastava, Abhishek Singh, Aashish Ranjan, Deepak Tugnawat, Yogendra Sen, Rahul Singh, Bhagwan Verma, Naveen Kumar Maheshwari, Harish Parmar, Narendra Verma, Kamlesh Sharma, Dharmendra Rathore, Anshika Malviya, Anant Bhan, John A Naslund

**Affiliations:** Sangath, 106, Good Shephard Colony, Kolar Road, Bhopal 462042, India; Sangath, 106, Good Shephard Colony, Kolar Road, Bhopal 462042, India; Sangath, 106, Good Shephard Colony, Kolar Road, Bhopal 462042, India; Sangath, 106, Good Shephard Colony, Kolar Road, Bhopal 462042, India; Sangath, 106, Good Shephard Colony, Kolar Road, Bhopal 462042, India; Sangath, 106, Good Shephard Colony, Kolar Road, Bhopal 462042, India; Sangath, 106, Good Shephard Colony, Kolar Road, Bhopal 462042, India; Sangath, 106, Good Shephard Colony, Kolar Road, Bhopal 462042, India; Sangath, 106, Good Shephard Colony, Kolar Road, Bhopal 462042, India; Sangath, 106, Good Shephard Colony, Kolar Road, Bhopal 462042, India; Sangath, 106, Good Shephard Colony, Kolar Road, Bhopal 462042, India; Sangath, 106, Good Shephard Colony, Kolar Road, Bhopal 462042, India; Sangath, 106, Good Shephard Colony, Kolar Road, Bhopal 462042, India; Sangath, 106, Good Shephard Colony, Kolar Road, Bhopal 462042, India; Department of Global Mental Health and Social Medicine, Harvard Medical School, 641 Huntington Avenue, Boston, Massachusetts 02115, United States

**Keywords:** community health worker, stress, ASHA, digital health and technology, mental health, Self Help Plus, stress alleviation methods, mixed methods evaluation

## Abstract

Psychological distress and risk of burnout among community health workers (CHWs) in low- and middle-income countries represent a serious global public health concern and threat to efficient health system functioning and resilience. This mixed methods study aimed to test the acceptability, feasibility and preliminary effectiveness of a digital adaptation of the WHO’s evidence-based Self-Help Plus (SH+) psychological intervention among CHWs, called Accredited Social Health Activists (ASHAs), in rural India. A total of 40 ASHAs, all women, were recruited from Sehore district, Madhya Pradesh, from October 2022 to March 2023. The intervention, a culturally adapted, digitized version of the WHO’s evidence-based SH+ intervention, called SAMBHAV, was delivered via smartphone app. Psychological distress was measured using the Kessler-10 at baseline, 6- and 12-week follow up. The System Usability Scale and Client Satisfaction Questionnaire-8 were used to assess usability and satisfaction with the digital intervention, respectively. Focus group discussions were used to assess acceptability. From baseline to 12-week follow-up, psychological distress levels significantly reduced (mean decrease of 2.5 points, *P* = .043), indicating improved psychological health and psychological distress management capacity. The intervention demonstrated favorable acceptability (mean = 20.45) and usability (mean = 69.31), though challenges related to user interface and app navigation were identified. Qualitative feedback supported these findings, with ASHAs describing the intervention as practical, easy to learn, and effective in reducing their psychological distress while empowering them to assist others in managing tension. These findings highlight that the WHO’s SH+ intervention can be adapted for different low resource contexts and tailored to meet the needs of specific target groups, specifically for alleviating psychological distress among frontline CHWs. Future research is needed to determine the benefits of scalable brief digital self-help interventions in promoting the well-being of frontline health workers and its resulting impacts on service delivery and health system functioning.

Key messages

- Psychological distress among community health workers (CHWs) in low- and middle-income countries threatens health system resilience. SAMBHAV, a digitally adapted version of the WHO’s Self-Help Plus (SH+) intervention, could alleviate stress among CHWs in rural India.
- The SAMBHAV intervention significantly reduced psychological distress among Accredited Social Health Activists over 12 weeks, demonstrating high acceptability, usability, and preliminary effectiveness in stress reduction despite some navigational challenges.
- Smartphone delivered SH+ interventions represent promising and scalable solutions to enhance CHWs’ well-being in resource-constrained settings.
- Further research is required to inform the large-scale adoption of SAMBHAV and assess its impact on public health service delivery and health system resilience.

## Introduction

Psychological distress and burnout among community health workers (CHWs) in low- and middle-income countries (LMICs) have become incessant global public health concerns (Dugani et al. 2018, Grossman-Kahn et al. 2018, Selamu et al. 2019, Alshawish and Nairat 2020). CHWs, who are predominantly women, are exposed to various stressors such as high infection risks, navigating gender hierarchies, and their communities’ cultural and religious beliefs, resource shortages, long working hours, heavy workloads, and inadequate compensation, all of which take a toll on their physical, mental, and emotional health (Selamu et al. 2017, Musoke et al. 2022, Shrivastava et al. 2023a, 2023b).

In the Indian context, Accredited Social Health Activists (ASHAs) represent the “spine” of the larger CHW ecosystem and constitute the world’s largest workforce of CHWs (Asthana and Mayra 2022). ASHAs are adult females typically residing in the villages where they work, and they receive minimal compensation according to incentive-based schemes (National Health Mission 2014). Importantly, ASHAs are involved in providing preventive, promotive and curative health services, including prenatal and postnatal care; supporting family planning; facilitating immunization; and screening and follow up for non-communicable disease care. Approximately more than one million ASHAs are working across nearly every state in India (individually serving a village of about 1000 community members) and acting as the “bridge” between the community and the public health system (Shet et al. 2018, Panda et al. 2019, Oswal et al. 2020). ASHAs play a vital role toward meeting universal health coverage goals; yet, their own mental health remains fragile and largely neglected (Tripathy et al. 2016, Shet et al. 2018, Muke et al. 2019, Scott et al. 2019, Oswal et al. 2020, Pulagam and Satyanarayana 2021, Manjunath et al. 2022, Shrivastava et al. 2023a, 2023b).

Moreover, public health emergencies, like the COVID-19 pandemic have only exacerbated the psychological burden impacting ASHAs in India, and CHWs more globally (Nepal et al. 2021, Gomes Fernandes Vieira-Meyer et al. 2023, Yahemba et al. 2023). This has potentially resulted in the worsening of mental health concerns in these CHWs and poses threats to overall health system functioning (Ghosh and Sengupta 2022, Menon et al. 2022). Some studies have reported high rates of depression, anxiety, and post-traumatic stress disorder (PTSD) among healthcare workers during the pandemic, with prevalence rates as high as 21.7% for depression, 22.1% for anxiety, and 21.5% for PTSD (Bayazit et al. 2022, Fernandez et al. 2022, Mohsin et al. 2024). Additionally, work-related fatigue and poor self-rated health have further compounded these challenges. While many countries implemented psychological crisis interventions for the general population in response to COVID-19, few of these efforts focused specifically on addressing the health of CHWs, especially mental health, in LMIC contexts (Yakubu et al. 2022, Ndulue et al. 2024).

Addressing the mental health challenges of CHWs like ASHAs is crucial for ensuring the long-term resilience and effectiveness of health systems globally. As efforts continue toward strengthening health systems and building a more resilient workforce, there is a need for mental health promotion interventions for CHWs. Importantly, such interventions need to be simple, scalable, and easily accessible within low-resource settings while considering the busy workload of CHWs. In addition, interventions that offer privacy and autonomy have the potential to address stigma and overcome barriers that prevent CHWs from seeking traditional mental health support. The Self-Help Plus (SH+) intervention, developed by the World Health Organization (WHO) as part of scaling up low-intensity psychological treatments, represents a potentially promising self-help intervention that could be deployed to support CHWs. Based on recommendations for stress management interventions (Tol et al. 2013), SH+ includes a five-part illustrated booklet and eight short audio sessions. It is designed to help people manage psychological stress and adversity without requiring a clinical diagnosis, making it suitable for large-scale use (Epping-Jordan et al. 2016). The theoretical underpinning of the SH+ is grounded in Acceptance and Commitment Therapy (ACT), a form of cognitive behavioral therapy that incorporates mindfulness and acceptance practices (Hayes et al. 2012, Karyotaki et al. 2023).

SH+ has been adapted for use in refugee populations across settings in Asian, African, and European countries, and recent studies have demonstrated its effectiveness in reducing psychological distress and sub-threshold depression and preventing the onset of mental disorders at longer term follow-up time points (Tol et al. 2020, Purgato et al. 2021, Lakin et al. 2023). Further investigation is crucial to confirm the acceptability and feasibility of SH+ for use among healthcare workers across different cultural settings given its affordability and open-source availability, as well as determining whether digital platforms can enable the delivery of this intervention.

In preparation for the current study, the SH+ intervention was adapted for use in rural India and digitized by employing participatory design workshops with ASHAs under a new intervention called the Stress Alleviation Methods for community-Based Health ActiVists (SAMBHAV) intervention development study (Shrivastava et al. 2023a, 2023b). The current study aims to contribute to bridging the gap in knowledge about the generalizability of the SAMBHAV intervention (i.e. the adapted digitized SH+ for ASHAs) in a rural Indian setting. This study involved a pilot mixed methods evaluation of the acceptability, feasibility, and preliminary effectiveness of the SAMBHAV intervention in addressing stress among ASHAs. Evidence generated from this study will enhance understanding of how the SAMBHAV intervention can be scaled up to address the elevated psychological distress impacting CHWs across India and in comparable low-resource settings globally.

## Materials and methods

This pilot study was conducted from October 2022 to March 2023 in Sehore, a rural district in Madhya Pradesh. With a population of over 75 million, of whom over 70% reside in rural areas, Madhya Pradesh represents one of India’s largest states. Relative to many other states in India, Madhya Pradesh ranks lower on the Human Development Index (Suryanarayana and Agrawal 2013). We employed a mixed-methods design to assess the feasibility, acceptability, and preliminary effectiveness of the SAMBHAV intervention. This involved collecting quantitative self-report measures, process indicators on user engagement and intervention completion, and supplemented by qualitative insights gathered from focus group discussions. Ethical approval for all study procedures was obtained from the Institutional Review Board of India and USA.

## Participants

Employing convenience sampling (Jager et al. 2017), we recruited ASHAs and ASHA facilitators (i.e. ASHAs with more years of experience who supervise and support ASHAs), who had previously participated in a large-scale study called ESSENCE, involving the use of a digital platform for training ASHAs in the delivery of a brief psychological intervention for depression (Tugnawat et al. 2024). To access the SAMBHAV intervention, we provided smartphones to participants for use throughout the study duration, because based on the ESSENCE program smartphone ownership data only half of the ASHAs own a smartphone. The smartphones had the learning app preloaded to access the SAMBHAV intervention content.

### Participant recruitment

The study data manager randomly selected 50 ASHAs (compared with the initial target of 40 ASHAs, with 10 additional ASHAs included to mitigate potential attrition) from the larger ESSENCE cohort. Study research assistants telephoned these ASHAs to brief them about the study and seek their verbal assent to participate. Of those who assented, small groups of 10–12 ASHAs were invited for a detailed orientation. The orientation lasted ∼4–5 h, and involved confirming participant details and eligibility, and providing an overview of the study objectives, giving a brief description of the intervention and outcome assessments, and explaining their expected role and estimated time commitment, and collecting written informed consent. ASHAs were informed that their participation was entirely voluntary, and that they could leave the study at any time without consequences. Following consent, participants completed baseline assessments using self-report questionnaires. All study participants received travel and daily allowance of INR 300 (approximately $4 USD) for completing study activities, which aligned with existing standard payments according to health system guidelines. Participants were not compensated for completing the 6-week SAMBHAV digital intervention. During the intervention delivery period, the study team provided remote support (i.e. phone calls and WhatsApp messages) to all participants to resolve any technical challenges encountered with navigating the app and accessing the digital content.

### Study intervention

We employed a stepwise participatory design process to culturally and contextually adapt the WHO’s SH+ intervention in Hindi language for use with ASHAs in Madhya Pradesh as part of the SAMBHAV intervention. This process is described in detail elsewhere (Shrivastava et al. 2023a, 2023b), ASHAs provided feedback, which led to refinements such as simplifying language, tailoring content to local norms, and ensuring materials were engaging and relevant to their daily experiences.

The core elements of SAMBHAV, which also means “possible” in Hindi, included instructional videos consisting of recorded lectures and PowerPoint presentations with voice-over. These short videos featured lectures that encompassed the fundamental content of SH+, along with role-play scenarios illustrating the application of various skills and techniques for managing psychological distress in everyday situations relevant to ASHAs. To promote participant engagement, various interactive assessment activities were also included, such as multiple-choice questions, drag-and-drop responses, and true/false questions related to the instructional content. The completed digital intervention content was uploaded to the Learning Management System (LMS), which is accessible via a smartphone application.

The digital content was supplemented with a printed version of the SAMBHAV manual which consisted of six modules that align with those in the digital intervention and original SH+ intervention content, serving as a supplementary resource for participants to reinforce concepts and revisit various psychological distress management techniques and strategies.

### Data collection and measures

We collected several process indicators directly from the LMS to determine feasibility and acceptability as reflected in participant completion and engagement with the SAMBHAV digital intervention content. Participants then completed follow-up assessments at 6- and 12-week intervals. Self-report questionnaires were collected to assess feasibility and acceptability and preliminary effectiveness and supplemented with qualitative focus group discussions.

#### Psychological distress

Our effectiveness outcome was psychological distress measured using the Kessler-10 (K10) (Kessler and Mroczek 1994) at 6- and 12-week follow-up, after baseline assessment. The K10 has demonstrated a Cronbach’s alpha of 0.74 (Patel et al. 2008), indicating moderately high internal consistency in Indian settings. The measure consists of 10 items about how the individual has been feeling in the last 30 days, with response options ranging from “None of the time” to “All the time.” The overall score ranges from 10, showing minimal distress, to 50, indicating severe distress (Andrews and Slade 2001).

#### System Usability Scale

We utilized the System Usability Scale (SUS) (Brooke 1996, Bangor et al. 2008) at the 6-week follow-up to measure usability of the SAMBHAV intervention. SUS is a 10-item, five-point Likert scale with response options ranging “Strongly Disagree” to “Strongly Agree” (Bangor et al. 2008).

### Client Satisfaction Questionnaire

The Client Satisfaction Questionnaire (CSQ-8) (Attkisson and Zwick 1982, Attkisson and Greenfield 2004) was administered at 6-week follow-up. The CSQ-8 measures participants’ overall satisfaction with the digital intervention. The items are rated on a four-point Likert scale (1 = dissatisfaction, 4 = high satisfaction). Total scores range from 8 to 32, with higher scores indicating greater levels of satisfaction.

## Statistical analysis

Stata-14 was used for quantitative data analyses. Descriptive statistics were used to examine the distribution of item scores, including the mean, and standard deviation (SD) with a 95% confidence interval (CI) for study assessments at each time point. To determine whether there were pre–post changes in psychological distress, we employed paired *t*-tests from baseline to 6- and 12-weeks follow up. We used an unadjusted repeated measures ANOVA (the statistical method used to test whether the means of the groups differ significantly by comparing between-group variance to within-group variance) to assess the effect of time on psychological distress; this was followed by an adjusted model accounting for participant demographic characteristics that could potentially have an impact on psychological distress (i.e. age, education level, work experience and family structure). The analysis included a sphericity correction to determine whether the reported *P*-value was accurate or potentially affected by a Type I error. Mixed effects models for repeated measures at baseline, 6- and 12-week time points were then conducted for the K10 scores while controlling for the baseline scores to explore intervention effects over time and account for the correlation among study participants and to capture variability within and between study participants. The CSQ-8 and SUS scores were summarized using descriptive statistics.

### Qualitative focus group discussions and thematic analysis

We collected qualitative insights through focus group discussions with a subset of 20 randomly selected participants to gather feedback on their experiences with the intervention, their perspectives on the usefulness and relevance of the content, and any recommendations for improving the intervention. We developed a semi-structured qualitative interview guide to facilitate the focus group discussions, which was accompanied by an *a priori* coding framework outlining broad themes relevant to digital intervention usability, acceptability, and feasibility within the context of rural India. The focus group discussions were audio recorded and transcribed and translated into English for analysis by study research assistants. A study co-investigator reviewed the raw transcripts and developed the coding framework, employing an iterative review process and allowing for discussion among team members to reach consensus. The coding framework is summarized in Table 1. The transcripts were manually coded using a deductive approach guided by the framework. Codes were then grouped into overarching themes.

**Table 1.** Coding framework

1. Acceptability of the intervention	1.1 Acceptability of the training program for ASHA workers
	1.2 Acceptability of the idea of stress reduction
	1.3 Acceptability of the content concepts
	1.4 Feedback on visual content- videos
	1.5 Feedback on audio content
	1.6 Feedback on text content
	1.7 Feedback on stories/narration
	1.8 Feedback on characters
	1.9 Feedback on quizzes, assessments
	1.10 Feedback on app/digital platform
2. Utility/usefulness of the intervention	2.1 Usefulness of the content
	2.2 Learning about stress/burnout
	2.3 Learning about techniques to reduce stress/burnout
3. Impact of the intervention	3.1 Impact on the personal life
	3.2 Impact on social life/perspectives
	3.3 Impact on professional life
	3.4 Impact on patients care
4. Challenges with the intervention and recommendations for improvement	4.1 Challenges in completing the training
	4.2 Difficulties in understanding the content and the concepts of content
	4.3 Difficulties in navigating the digital content
	4.4 Any technical difficulties
	4.5 Suggestion for improvement

## Results

Table 2 summarizes socio-demographic characteristics for all 40 participating ASHAs. Nearly half (*N* = 19; 47.5%) were aged between 32 and 36 years. Most (55%) had education from grades 10–12; while 27.5% pursued higher education beyond the 12th grade. A majority (62.5%) had at least 3 years of work experience and nearly all (95%) were married. All study participants completed all modules of the SAMBHAV intervention.

**Table 2.** Socio-demographic characteristics of participants

*N* = 40		
Characteristic	*N*	Percentage
Age group		
26–31	7	17.5
32–36	19	47.5
37–41	7	17.5
42–45	7	17.5
Education in grades		
8–9	7	17.5
10–12	22	55
Higher studies	11	27.5
Work experience (years)		
1	3	7.5
2	10	25
3	25	62.5
4	2	5
Marital status		
Married	38	95
Widowed	2	5
Family type		
Joint	19	47.5
Nuclear	21	52.5
Head of a family		
Father-in-law/mother-in-law	10	25
Husband	26	65
Self	4	10
Religion		
Hindu	39	97.5
Muslim	1	2.5
Caste		
General	2	5
OBC	24	60
SC	13	32.5
Others	1	2.5
Training modules completion (ASHA gov. mandated)		
1	1	2.5
4	1	2.5
5	7	17.5
6	6	15
7	25	62.5
Total	40	100

*N*, sample size; OBC, other backward classes; SC, scheduled caste; ASHA, accredited social health activist; gov., government.

### Psychological distress

K10 scores decreased from baseline (mean = 21.83; SD = 6.93) to 6-weeks (mean = 20.90; SD = 5.26) and 12-weeks (mean = 19.33; SD = 5.94), as summarized in Table 3. Paired *t*-tests showed no significant change from baseline to 6-weeks (*P* = .433), but a significant decrease in K10 scores from baseline to 12-weeks (*P* = .043).

**Table 3.** Psychological distress measured using the K-10 at baseline and at 6- and 12-weeks follow up

Variable	Mean	SD	95% CI	*N*
K-10 at baseline	21.83	6.93	19.6–24.04	40
K-10 at 6-weeks	20.90	5.26	19.2–22.6	40
K-10 at 12-weeks	19.33	5.94	17.4–21.2	40

K-10	Paired *t*-test	*P*-value	DF
Baseline to 6-weeks	0.793	.433	39
Baseline to 12-weeks	2.091	.043	39

K-10, Kessler psychological distress scale (10-item version); SD, standard deviation; CI, confidence interval; *N*, sample size; DF, degrees of freedom; *P*-value, probability value (significance level for the test statistic).

As shown in Table 4, in the unadjusted repeated measures ANOVA, the within-subjects effect of time approached significance, *F* (2,78) = 3.08, *P* = .0517. Given that Mauchly’s test indicated a potential violation of sphericity, we applied Greenhouse–Geisser (ɛ = 0.6782) and Huynh–Feldt (ɛ = 0.6941) corrections, adjusting the *P*-values to .0728 and .0716, respectively. These corrections show that the time effect approached statistical significance at the conventional alpha level of 0.05 when accounting for sphericity violations. In the adjusted model, the time effect remained significant, showing that K10 scores decreased over time [coefficient = −1.25, SE = 0.47, 95% CI (−2.18, −0.32), *P* = .008] after adjusting variables like score at baseline, and participant demographic characteristics (see Table 4).

**Table 4.** Unadjusted ANOVA table for within-ASHA effects with sphericity correction along with the mixed effect model

Unadjusted						
Source of variation	Partial SS	DF	MS	*F*	Prob > *F*	
Within-ASHAs: time	127.82	2	63.91	3.08	0.0517	
Residual	4326.15	117	36.98			
Total	4453.97	119	37.43			

Sphericity correction table						
Effect	Source	Huynh–Feldt epsilon value	Greenhouse–Geisser epsilon value	Adjusted *P* (Huynh–Feldt)	Adjusted *P* (Greenhouse–Geisser)	Adjusted *P* (Box)
Time	2	0.6941	0.6782	0.0716	0.0728	0.0872

Mixed effect model for repeated measures ANCOVA						
Source of variation	Coefficient	SE	*Z*	*P*-value	95% CI	
Time	−1.25	0.47	−2.64	.008	−2.18	−0.32
Score at baseline	0.83	0.09	9.05	.000	0.65	1.01
Age	0.02	0.14	0.15	.88	−0.25	0.29
Work experience (years)						
2	−0.80	2.36	−0.34	.736	−5.43	3.83
3	−0.78	2.21	−0.35	.723	−5.11	3.54
4	−2.21	3.30	−0.67	.503	−8.68	4.26
Family type						
Nuclear	−0.19	1.27	−0.15	.884	−2.67	2.30
Education (grades)						
10–12	−0.01	1.59	0	.996	−3.13	3.11
Higher studies	−0.47	1.82	−0.26	.797	−4.04	3.10
Intercepts	6.01	3.41	1.76	.078	−0.68	12.69

SS, sum of squares; DF, degrees of freedom; MS, mean square; *F*, *F*-statistic; Prob > *F*, probability value for *F*-test; SE, standard error; *Z*, *Z*-statistic; CI, confidence interval; ANCOVA, analysis of covariance.

### System Usability Scale

For the SUS, the mean score at 6-week follow up was 69.31 (SD = 11.51) with a 95% CI (65.63, 72.99). A score of 69.31 indicates that participants found the digital intervention to be reasonably usable. However, as the score is close to the threshold of acceptability, it suggests that while the system is usable, there could be scope to improve the user experience further.

### Client Satisfaction Questionnaire

The mean CSQ-8 score at 6-week follow up was 20.45 (SD = 2.28) with a 95% CI (19.72, 21.18). A mean score of 20.45 suggests a moderate level of satisfaction among participants.

## Qualitative findings

Qualitative feedback gathered from ASHAs was grouped into four primary themes aligned with the *a priori* coding framework (see Table 1). Representative quotes from participants were included for each theme and summarized in the sections that follow.

### Theme 1: acceptability of the intervention

This first theme focuses on ASHAs overall perceptions of the intervention, with specific attention to how each type of content—such as videos for their visual appeal, audio for ease of access, and other materials for practical guidance—was received. Overall, ASHAs expressed positive experiences using the intervention:

Everyone should get to know about this intervention and learn it because, no one is stress free, everyone faces stress somehow. (ASHA, 33 years with postgraduate education, 2 years of work experience, and living in a joint family)

ASHAs commented on how the intervention content could also be relevant for both genders, given the pressing need for stress reduction:

In the household, everyone faces stress. Whether it's male or female, everyone experiences stress. But because of this training, now we know that yes, we do have stress, but we have learned how to handle and manage it. We have learned how to get rid of stressful situations. This is what this training has taught us. We have understood very well, what we can do when we face stress. We have gained the strength to face our stress and tensions bravely. (ASHA, 36 years with high school education, 3 years of work experience, and living in a nuclear family)

Regarding the layout and presentation of the content, many ASHAs expressed appreciation for the storytelling methods used to convey key concepts, finding the narratives emotionally engaging. One ASHA mentioned the comparison of human nature to clouds and liked the metaphor of linking moods to changing weather, as she could relate this to her own emotions:

To reduce tension, there was an example given where they explained our nature with clouds. Clouds have to go through many weather conditions, but they don't change; they stay strong even in storm-like conditions. They compared our mood with the weather. That part I liked the most, and I could apply it to myself. I understood it very well and really liked it. (ASHA, 34 years with high school education, 3 years of work experience, and living in a nuclear family)

Some ASHAs found the content of the intervention like tea-drinking with family, to be enjoyable and simple:

There were two simple exercises as part of this training I really enjoyed doing. One exercise involved the activity of drinking tea, the other exercise centered around playing or reading with kids, emphasizing the importance of giving full attention to the activity. We have to notice the colors of toys, engage in the game wholeheartedly, and genuinely enjoy the experience. This was the exercise of doing focus. (ASHA, 45 years with high school education, 3 years of work experience, and living in a nuclear family)

Many ASHAs appreciated seeing characters that resembled their own lives, challenges, and emotions, which made the content feel personal and engaging:

In that video, they explained the reasons for stress and how to reduce it. There were two stories of women depicted. One of the stories was about Meena, who was experiencing stress. She had a small family with two kids and a husband who worked at a brick kiln. Meena herself was an ASHA worker. On top of her family responsibilities, her mother-in-law was elderly and terminally ill, which added to her struggles. She had to balance her household affairs and work, taking care of her kids and managing household responsibilities. It was a relatable story. (ASHA, 33 years with postgraduate education, 2 years of work experience and living in a joint family)

Overall, the digital platform was perceived as both convenient and empowering for learning the intervention, allowing participants to refer to the content whenever they wanted, at their own pace. ASHAs commented on the use of interactive quizzes on the platform and mentioned that it made learning easier and enjoyable:

If someone teaches using book language, it may not be easily understood, but learning through (interactive) activities can be more effective. For example, after explanation of concept and then we get engaged in activities (Quizzes), I grasped it quickly. (ASHA, 33 years with postgraduate education, 2 years of work experience, and living in a joint family)

One ASHA also highlighted the utility of the digital platform, stating that she could repeatedly access the app to reinforce her learning, emphasizing the platform's value in enabling self-paced and flexible engagement with the material.

This app is excellent for learning and understanding content easily and effectively. The best part is, if you don't understand a question and answer it incorrectly, you can try it again after re-watching the video and learning again. It makes me feel like a kid again, and I had a lot of fun doing this activity. (ASHA, 42 years with high school education, 3 years of work experience, and living in a joint family)

### Theme 2: utility/usefulness of the intervention

This second theme pertains to whether ASHAs found the intervention content practical, engaging, and relevant to their experiences. This included determining how the intervention helped ASHAs learn about stress and burnout, specifically the causes, symptoms, and the ways these issues impact their personal and professional lives, as well as learning about stress reduction techniques. Many ASHAs commented on the usefulness of the content and how the intervention increased their awareness:

The training was designed in such an easy way that before taking it, I didn't even realize that I was in tension and stress. However, after completing the training, I became aware of the tension I was facing. It was very helpful for me to cope with my situation and the associated tension. (ASHA, 45 years with high school level education, 3 years of work experience, and living in a nuclear family)

Another ASHA explained how exercises help her manage stressful situations and are useful in preventing her from being consumed by unpleasant thoughts and focusing on the moment to feel at ease:

It's like this, suppose we are alone and having unpleasant thoughts. Our mind becomes filled with those unpleasant thoughts. At that moment, if we are sipping tea, we should only focus on the tea itself. It might sound crazy or funny, and I also felt the same at the beginning, wondering why I was doing such a seemingly stupid thing. However, by practicing this exercise and fully immersing in the act, we can forget about those unpleasant thoughts. It's like to changing our environment when we have arguments or fights at home. (ASHA, 42 years with high school education, 3 years of work experience, living in a joint family)

Similarly, another ASHA described how the techniques help overcome “emotional storms,” reflecting the connection to the metaphor linking mood and weather:

The same (concept) applies to the sky exercise. For example, we can watch birds and listen to their humming, or focus on other soothing sounds like temple aarti or chants. By directing our attention to these pleasant things during moments of emotional storm, we can alleviate the impact of those unpleasant thoughts. It is for our emotional storm. (ASHA, 33 years with higher education, 3 years of work experience, and living in a nuclear family)

Other ASHAs described how the activities have become a part of their daily routine, providing an opportunity to practice the exercises to relax after a long day at work:

I feel that when I return home after a long day of doing surveys and completing other tasks, I used to have tea around 4pm. But now, when I drink tea at 4 pm, I practice the exercises that were taught in the video. During at least that time, I feel relaxed by doing the exercise along with having tea. (ASHA, 33 years with postgraduate education, 2 years of work experience, and living in a joint family)

Several ASHAs also expressed that learning about stress-relief techniques, such as specific exercises, was beneficial and empowering:

In this training, I learned that when you experience continuous stress for 10 or 15 days, it's essential not to isolate yourself and dwell on your thoughts for an extended period. If you have kids or someone at home, try to spend time with them. Whenever I feel overwhelmed or stressed, I choose to be with kids or friends. I engage in conversations with my friends, listen to their problems, and shift my focus from my own worries to theirs. (ASHA, 32 years with higher school education, 2 years of work experience, and living in a joint family)

Another ASHA commented that understanding how to actively address and release tension was a transformative experience:

I can't even express how much this has helped me. The explanation of stress and how to overcome it was done in a very relatable manner. Ever since we started taking this training, we have noticed many positive changes in ourselves. My anxiety and irritability issues have reduced. (ASHA, 42 years with high school education, 3 years of experience, and living in a joint family)

### Theme 3: impact of the intervention

The third theme captures ASHAs’ perceptions about the impact of the intervention on their personal, social, and professional lives as well as the care they provide to their patients. Most ASHAs reported experiencing positive changes in themselves, and described feeling better, with improvements in various aspects of their lives. Some mentioned experiencing an uplifted mood, while others noted enhanced well-being and better overall mental health:

There have been changes in me. I feel a difference in how I speak. Before this training, I used to experience a lot of stress and struggled to cope with challenging situations. But, since this training, I have gained the strength to face any and every situation. (ASHA, 32 years with higher school education, 2 years of work experience, and living in a joint family)

Several ASHAs shared their personal stories of overcoming stress while applying learning from the intervention.

I want to share my own story. In my home, we used to have a tense and argumentative environment, and I would stay silent, sitting in the corner because of that atmosphere. But now, I can confront that situation. Since I have received this training, I have gained courage. (ASHA, 32 years with higher school education, 2 years of work experience, and living in a joint family)

Some ASHAs also described how the content helped them feel more empowered in navigating challenging personal situations.

Let me give an example from my own experience. Earlier, when someone used to have a conversation with me, I would feel annoyed and angry inside. However, now I consciously try to stop that anger and control myself. I have become better at managing my anger. (ASHA, 32 years with high school education, 2 years of work experience, and living in a nuclear family)

While other ASHAs expressed feeling positively impacted from the exercises:

By doing these exercises, I feel energized, refreshed and much more energetic to take on any task. (ASHA, 33 years with higher school education, 3 years of work experience, and living in a nuclear family)

One ASHA mentioned that she believes this training can benefit other women experiencing stress and would recommend this training to others:

By doing these exercises, we can reduce our tension, we can suggest these exercises with other women who feel stressed. They can also benefit from these exercises. (ASHA, 33 years with post graduate education, 2 year of work experience, and living in a joint family)

When asked if they noticed any changes in their workplace after completing the training, one ASHA remarked:

My anger has also reduced. I used to get angry about every matter at workplace, whether it was something going wrong or being scolded by madam or sir (senior officials). But now, it has reduced. (ASHA, 29 years with graduate education, 2 years of work experience, and living in a nuclear family)

While another ASHA expressed feeling better able to take on new tasks as part of her work:

I am now able to handle my work, household chores, and field work. Additionally, I don't feel tense about taking on new tasks anymore. I believe I can manage, and I am indeed managing my work now. (ASHA, 45 years with graduate education, 3 years of work experience, and living in a nuclear family)

Several ASHAs also mentioned that the intervention content has been beneficial in enhancing their professional knowledge, as new modules on mental health have recently been introduced in the government’s routine training interventions. Moreover, they emphasized that the intervention provided valuable insights and practical skills that align with these updated modules, supporting their ability to integrate mental health concepts into her work more effectively including interactions with patients to provide better support.

Questions related to mental health issues have been added to the NCD (non-communicable disease care) form for us to fill. However, we cannot directly ask the patient, ‘Do you have a mental health issue?’ But thanks to the training, we have learned about the symptoms and how to recognize them. This training has been very helpful for us in this regard. (ASHA, 42 years with high school education, 3 years of work experience and living in a joint family)

Some ASHAs mentioned that they have started applying the knowledge and techniques acquired from the intervention in their interactions with patients.

In this training, we learned how to handle various people in different ways. For example, when we visit a self-help group and talk to the women there, we understand that everyone is not the same. So, we keep in mind that people are different and approach them accordingly. (ASHA, 42 years with high school education, 3 years of work experience, and living in a joint family)

Considering the impacts of the COVID-19 pandemic, many ASHAs felt that having access to such an intervention sooner could have improved their experiences, with some sharing their personal struggles related to burnout and mental health:

This training seems relatable to me. I feel that sometimes what is explained here aligns with me and my own experiences. Speaking honestly, during the COVID time, we all went through challenging times, and many of us experienced depression. I wish we had discovered this training during that period; it could have helped us cope with many things. The training contains very useful information; we just need to learn them. (ASHA, 42 years with high school education, 3 years of work experience, and living in a joint family)

Another ASHA shared her experience of suicidal ideation, expressing that the training provided her with valuable support during a challenging time in her life. She emphasized that such interventions could be beneficial for many young girls and women facing similar struggles, helping them manage their stress and navigate difficult circumstances.

This training is really good and highly relevant to our work. We have no problems with it; there is nothing in this training that we don't like. The only thing that stood out was how exceptionally beneficial it is. It proves to be effective not only for us but also for the people we are connected to. Particularly for women and adolescent girls, this training is superb. I can personally relate to it and understand its impact. Sometimes, I think that receiving this training was a life-changing experience for me. Without it, a person like me might have hanged myself and could have committed suicide. But now I feel how much I like this. (ASHA, 32 years with higher school education, 2 years of work experience, and living in a joint family)

### Theme 4: challenges with the intervention and recommendations for improvement

ASHAs highlighted a few challenges they faced in using the intervention, including not having enough time to complete the program and finding some content difficult to follow. For instance, one ASHA remarked:

I haven’t faced any difficulties, only the thing that in family and work I haven’t got time, so only the time constraint I had, I could not take this training on my desired time I had to take it during night. (ASHA, 32 years with high school education, 2 years of work experience, and living in a nuclear family)

While many ASHAs enjoyed the video-based content and use of role-plays, feedback on the audio content was mixed, with some ASHAs commenting that the audio content alone was less engaging and required the addition of visualizations to enhance their learning experience and maintain their interest.

I feel that I learn better by watching videos rather than just listening to audio. When I watch a video, the information imprints on my mind and stays with me for a longer time. But, when I only listen to audio, I tend to forget about it quickly. (ASHA, 33 years with postgraduate education, 2 years of work experience, and living in a joint family)

Some ASHAs also expressed difficulties with understanding certain concepts within the intervention content including the difficulties in navigation and delays in opening the app and content.

There were a few parts where I felt I couldn't understand the questions. In a few instances, I found it difficult to comprehend the questions being asked. As a result, I made mistakes in those questions. Another issue we encountered was that after attempting the questions, the process of reviewing them wasn't clear to me. Additionally, there were times when it wasn't clear whether we had completed the course or not. (ASHA, 32 years with graduate education, 2 years of work experience, and living in a nuclear family)

ASHAs also offered some suggestions to improve the intervention content, such as expanding the content covered in the written manual to more closely align with the digital content:

If there were questions given in the book, it would have been better. Then, perhaps, we could have understood the concepts more easily. Currently, the book only contains stories. (ASHA, 42 years with high school education, 3 years of work experience, and living in a joint family)

Other ASHAs also commented on technical challenges, such as videos not being able to load, further highlighting the need for simplification of the digital platform and reducing the content size to ensure smooth operation across rural settings:

There was only one problem—sometimes the videos wouldn't open or would play slowly. sometimes they would open and then proceed. It seems like there were technical issues which should be resolved. (ASHA, 33 years with postgraduate education, 2 years of work experience, and living in a joint family)

## Discussion

This pilot study involved a preliminary evaluation of an adapted and digitized version of WHO’s evidence-based SH+ intervention, tailored to address psychological distress experienced by ASHAs in rural India. Specifically, the original SH+ intervention was adapted to the local culture, context, and language, and was adapted for digital delivery in a self-paced format via a smartphone app. This pilot study offers valuable insights regarding the feasibility and acceptability of the SAMBHAV intervention for addressing the mental health burden faced by ASHAs due to work-related psychological distress and risk of burnout, which was further exacerbated by the pandemic. Importantly, we observed notable reductions in psychological distress levels at the 12-week follow-up (mean decrease of 2.5 points, *P* = .043), suggesting improved psychological health and an improved capacity to manage psychological distress. These preliminary findings show that the SH+ intervention, after adaptation to alternative contexts and settings, can achieve similarly beneficial outcomes for frontline health worker (Tol et al. 2020, Li et al. 2024).

The combination of both quantitative and qualitative findings highlights the feasibility and acceptability of the adapted SH+ intervention, as reflected by higher scores on the SUS and CSQ-8 measures, as well as promising feedback and perspectives shared by participating ASHAs. For instance, ASHAs commented on the value of the content for their everyday lives, and the usefulness of the intervention. This suggests that the techniques covered were straightforward and easy to learn with minimal guidance, making this a suitable intervention for individuals with varying levels of education or limited familiarity with mental health practices. Many ASHAs also emphasized the benefits of the intervention, viewing it as not only empowering for managing their own psychological distress but also equipping them with the knowledge and confidence to share these techniques with others. ASHAs also commented on the intervention's usefulness in addressing distress for ASHAs and for other women serving in caregiving roles. This reflects the burden of managing multiple responsibilities, which often leads to psychological distress and emotional strain, and suggests that the adapted techniques from the SH+ content hold potential to equip women in rural low-resource settings with practical strategies to manage psychological distress.

Despite these promising findings, several ASHAs provided insights about technical challenges that they experienced, such as the complexity of the user interface, and the navigational ease required to access the digital content. This aligns with similar findings where primary health workers have shown reasonable satisfaction with digital interventions, yet have indicated the need for better user-friendliness or experiencing information overload (Tol et al. 2018, Brog et al. 2022). As part of intervention delivery, we leveraged popular digital platforms like WhatsApp to respond to challenges ASHAs faced concerning navigating the digital app and content, ensuring efficient delivery of standardized content without requiring extensive human resources. During the COVID-19 pandemic, many frontline healthcare workers expressed a demand for direct and easily accessible mental health support. Feedback from ASHAs further underscored this need, reflecting their desire for interventions that were both simple and readily available. As ASHAs increasingly gain access to smartphones as part of their work, through government initiatives, this adapted SH+ intervention holds promise for scale up in conjunction with other digital modalities and learning initiatives (Bashingwa et al. 2021).

Our promising findings of reduction in psychological distress, as reflected by improved K-10 scores over time, align with prior evaluations of the WHO’s SH+ intervention. For instance, in a randomized control trial enrolling refugees in Uganda, the SH+ intervention contributed to significant reductions in psychological distress observed at 12-week follow-up when compared with a usual care control condition (Tol et al. 2020). In a subsequent trial among Syrian refugees in Turkey, individuals receiving the SH+ intervention were significantly less likely to experience a mental disorder at 6-month follow up when compared with an enhanced usual care condition (Acarturk et al. 2022). It is noteworthy that in each of these prior studies, the SH+ intervention was delivered in a group format, whereas our adapted version of the SH+ intervention was self-directed and delivered via a smartphone app. These significant adaptations to the intervention were necessary to accommodate the heavy workload for ASHAs and to offer a potentially scalable psychological distress reduction intervention that could easily be integrated into their routine, without adding more burden. Therefore, the initial benefits observed in our pilot study appear to replicate the findings from several prior randomized controlled trials of the SH+ intervention such as mentioned above, while also demonstrating the utility of an adapted version of the intervention.

Despite these positive outcomes, several limitations should be considered that could affect the interpretation and generalizability of these findings. This study employed convenience sampling, primarily recruiting participants from the existing ESSENCE project. The specific selection of ASHAs may limit the generalizability of the results to the broader population of healthcare workers who may show varying digital literacy. Providing smartphones to ASHAs to access the SAMBHAV content could bias our study's findings and pose sustainability challenges, potentially acting as a reward that influenced participants’ involvement and engagement. Future studies should consider diverse strata of participants and longer follow-up periods to assess the sustained impact of the intervention across different groups. To facilitate the successful implementation of the SAMBHAV intervention within existing public healthcare system, further research is essential. This includes the evaluation of training and support for primary healthcare workers, financing mechanisms, organizational factors that could impede scalability, and cost-effectiveness of the intervention. Particularly, its cost-effectiveness aspect could inform how the impact of this intervention is associated with better work performance, better service delivery and reduced psychological distress-induced absenteeism among ASHAs and other related CHW cadres. Further, while this brief psychological distress reduction intervention showed promising impact, this intervention does not represent a substitute for improving the working conditions for ASHAs through equitable compensation and addressing gender inequalities and existing health system hierarchies that are the source of many psychological distress of affecting this critical workforce (Shrivastava et al. 2023a, 2023b).

## Conclusion

This pilot study highlights that SAMBHAV, an adapted digital version of the WHO’s evidence-based SH+ intervention, offers a promising approach for addressing psychological distress among frontline women healthcare workers in rural India. A distinctive aspect of the SAMBHAV intervention is its ability to be delivered via smartphone, making it accessible in contexts where mental health professionals are sparse and where transportation barriers make in-person interventions impractical. Unlike traditional psychological interventions that rely on specialist providers and in-person contact, SAMBHAV offers a remote self-help approach suitable for low-resource settings, where several challenges like stigma and lack of awareness surrounding mental health often prevent individuals from seeking support. Our study highlights that the SH+ intervention can be adapted for different contexts and settings, and for use in a different target population, reflecting the universal relevance of proven psychological distress management techniques that draw from ACT. This brief self-help psychological intervention appears both acceptable and effective, and with the use of smartphone delivery, can potentially be scaled up for broad use among ASHAs across India, or adapted for use among cadres of frontline healthcare workers in other low-resource settings. Future implementation research and cost-effectiveness analyses are warranted to guide the uptake and integration of psychological distress reduction techniques for CHWs.

## Data Availability

Data cannot be shared for ethical/privacy reasons.The data underlying this article cannot be shared publicly due to [ the privacy of individuals that participated in the study and some of them having lived experiences]. The unidentifiable data will be shared on reasonable request to the corresponding author.

## Reflexivity statement

The authors include two women and thirteen men, spanning multiple levels of seniority. Thirteen authors are based in Bhopal, Madhya Pradesh, India, while one author is from the USA. The authors come from backgrounds in public health and research. Some early-career researchers have substantial ground-level experience in the field of mental health. All authors have experience in conducting qualitative analysis and research in mental health services.
